# The Evolutionary Pattern and the Regulation of Stearoyl-CoA Desaturase Genes

**DOI:** 10.1155/2013/856521

**Published:** 2013-11-07

**Authors:** Xiaoyun Wu, Xiaoju Zou, Qing Chang, Yuru Zhang, Yunhai Li, Linqiang Zhang, Jingfei Huang, Bin Liang

**Affiliations:** ^1^State Key Laboratory of Genetic Resources and Evolution, Kunming Institute of Zoology, Chinese Academy of Sciences, Kunming, Yunnan 650223, China; ^2^Key Laboratory of Animal Models and Human Disease Mechanisms of the Chinese Academy of Sciences and Yunnan Province, Kunming Institute of Zoology, Kunming, Yunnan 650223, China; ^3^Department of Life Science and Biotechnology, Kunming University, Kunming 650214, China

## Abstract

Stearoyl-CoA desaturase (SCD) is a key enzyme that converts saturated fatty acids (SFAs) to monounsaturated fatty acids (MUFAs) in the biosynthesis of fat. To date, two isoforms of *scd* gene (*scd1* and *scd5*) have been found widely existent in most of the vertebrate animals. However, the evolutionary patterns of both isofoms and the function of *scd5* are poorly understandable. Herein, we aim to characterize the evolutionary pattern of *scd* genes and further predict the function differentiation of *scd* genes. The sequences of *scd* genes were highly conserved among eukaryote. Phylogenetic analysis identified two duplications of *scd* gene early in vertebrate evolution. The relative rate ratio test, branch-specific *dN/dS* ratio tests, and branch-site *dN/dS* ratio tests all suggested that the *scd* genes were evolved at a similar rate. The evolution of *scd* genes among eukaryote was under strictly purifying selection though several sites in *scd1* and *scd5* were undergone a relaxed selection pressure. The variable binding sites by transcriptional factors at the 5′-UTR and by miRNAs at 3′-UTR of *scd* genes suggested that the regulators of *scd5* may be different from that of *scd1*. This study promotes our understanding of the evolutionary patterns and function of SCD genes in eukaryote.

## 1. Introduction

Stearoyl-CoA desaturase (SCD) is an intrinsic membrane protein that binds to the endoplasmic reticulum (ER), composed of four transmembrane domains [1–3]. SCD is the rate-limiting enzyme that introduces the first cis-double bond at the delta-9 position of saturated fatty acids (SFAs) to thereby generate monounsaturated fatty acids (MUFAs) [4], which are major substrates for biosynthesis of polyunsaturated fatty acids (PUFAs) and complex lipids such as triglycerides, phospholipids, cholesterol esters, and wax esters being as energy storage, components of biological membrane, and signaling molecules. The ratio of unsaturated fatty acids to saturated fatty acids plays a vital role in cell signaling and membrane fluidity, in which imbalance of this ratio is often associated with diseases like diabetes, cardiovascular diseases, fatty liver, cancers and stresses resistance, and so forth [5].

The* scd* genes are universally present in living organisms. The number of *scd* genes varies from one to five, which are generally called *scd1, scd2, scd3, scd4, *and *scd5* in different organisms [4, 6], but with other distinct names in invertebrates such as *fat-5*, *fat-6*, and *fat-7* in *Caenorhabditis elegans *[7–9] and *ole1* in *Saccharomyces cerevisiae *[10]. The yeast genome contains only *ole-1* gene encoded SCD, and *ole-1* mutant requires unsaturated fatty acids for growth [10]. The desaturase of *C. elegans* FAT-5, FAT-6, and FAT-7 displays substrate preferences, in which both FAT-6 and FAT-7 mainly desaturate stearic acid (18 : 0) and have less activity on palmitic acid (16 : 0). On the contrary, FAT-5 desaturates palmitic acid (16 : 0) but has nearly undetectable activity on stearic acid (18 : 0) [7]. The evolutionary history revealed that the *scd* genes in vertebrates could be distinctly classified into *scd5* type [3, 6, 11] and *scd1* type including its homologs *scd2, scd3*,* and scd4* [6, 12]. The divergence of *scd1* and *scd5* genes occurred early in vertebrate evolution due to the whole genome duplication (2R) [6]. However, the *scd* genes may have distinct fates after gene duplication event. It is unknown whether one *scd* evolved faster and acquired new function more rapidly than the other, and whether the selective patterns on both *scd* genes were similarly changed following the duplication. 

Interestingly, though the enzymes of *scd* genes display similar delta-9 desaturation activity [4], the expression pattern of *scd1* and *scd5* is variable that *scd1* is ubiquitous, but *scd5* is mainly in the brain and pancreas even in different species [3, 6, 11], implying that the regulation of *scd1* and *scd5* expression and biological function may be distinct. The promoter region of *scd1* contains many consensus binding sites for numerous transcription factors, for example, SREBP1, LXR, PPAR*α*, C/EBP-*α*, NF-1, NF-Y, and Sp1 [13]. However, it is unclear whether *scd5* contains similar or completely different consensus binding sites with *scd1*. Meanwhile, it is completely unknown that the 3′-UTR of *scd1* and *scd5* that may also contain similar or different target sites of microRNAs regulating their expression.

Therefore, to address the above questions, we compared the sequence characteristics of *scd* paralogs and then reconstructed the phylogenetic trees of *scd* genes in eukaryote species to determine the evolutionary history of *scd* genes. We used the relative rate ratio test, branch-specific *dN*/*dS* ratio tests, and branch-site *dN*/*dS* ratio tests to analyze the evolutionary forces after gene duplication. Furthermore, we characterized the binding sites by transcript factors in the 5′-UTR and the target sites by microRNAs in the 3′-UTR of *scd1* and *scd5* genes to investigate the regulation mechanisms of both *scd* genes.

## 2. Material and Methods

### 2.1. SCD Homologs BLAST, Sequence Alignment, and Phylogenetic Analysis

SCD homologs were retrieved by key word “Stearoyl-CoA desaturase” from NCBI GenBank (http://www.ncbi.nlm.nih.gov/genbank/) and Ensemble genome database (http://asia.ensembl.org/index.html). In addition, the sequences of human SCD proteins were used to blast available genomes from NCBI GenBank and Ensemble database. Eventually, 73 *scd* nucleotide sequences from 39 representative eukaryote species were retrieved (see Table S1 in the Supplementart Material available online at http://dx.doi.org/10.1155/2013/856521). Sequence alignment of 73 *scd* nucleotides was performed with MegAlign implemented in DNAStar 6.0 software package (DNASTAR, Madison, USA) and then was confirmed visually by BioEdit 7.0.9 [14]. The ambiguous regions of alignment were discarded and eventually 720 nucleotide bases were obtained.

Phylogenetic tree was reconstructed based on the full alignment of 73 sequences by using Maximum Likelihood (ML) analysis in PHYML [15] and approximately Maximum Likelihood (ML) analysis in FastTree 2.1.3 [16]. The yeast *scd* ortholog, *ole1*, was used as the outgroup to root the tree. For ML analysis, supports for nodes among branches were evaluated using nonparametric bootstrapping [17] with 1000 bootstrap replications. For FastTree 2 analysis, a heuristics search strategy was employed with an estimated rate of evolution for each site (the “CAT” approximation), minimum-evolution subtree-pruning regrafting (SPRs), and maximum-likelihood nearest-neighbor interchanges (NNIs). The local support values were provided based on the Shimodaira-Hasegawa (SH) test [18, 19].

To evaluate the evolutionary conservation of the SCD1 and SCD5, the amino acid sequences of SCD1 and SCD5 of 11 model organisms including human, rhesus monkey, mouse, rat, tree shrew, zebrafish, *Drosophila melanogaster*, and *C. elegans* were retrieved and then aligned using Muscle (http://www.ebi.ac.uk/Tools/msa/muscle/), followed by manual adjustment with BioEdit 7.0.9 [14]. Additionally, a Neighbouring-Joining (NJ) tree was reconstructed with the amino acid sequences of SCDs from human, rhesus monkey, mouse, rat, tree shrew, and *C. elegans* by MEGA 4.0 [20] using amino acid p-distance model. Support for nodes among branches was evaluated using nonparametric bootstrapping [17] with 1000 bootstrap replications.

### 2.2. Regulation Prediction in 3′-UTR and 5′-UTR of *scd* Genes

Searching for the transcription factor-binding sites (TFBS) in the 5′-UTR of *scd* genes was carried out based on the positive effectors of transcription in the promoter region of *scd1* from human, mouse, and chicken [13]. The length of 5′-UTR for this analysis was about 2500 bp upstream of the translational start sites of *scd5* gene. The TFBSs were estimated by Match 1.0 with the TRANSFAC database v. 6.0 and Promo with TRANSFAC database v. 8.3 [21, 22]. The cut-off parameters were set as 0.75 for the core similarity and 0.85 for matrix similarity in Match 1.0 analysis. In Promo analysis, the species of factor and site were only constrained to animals. MultiSearchSite was used to search for binding sites sharing 15% maximum matrix dissimilarity rate in the promoter sequences of human, rhesus monkey, tree shrew, and chicken. 

The microRNA targets sites in the 3′-UTR region of *scd* genes were predicted by using TargetScan release 6.2 (http://www.targetscan.org/). The lengths of the 3′-UTR region of *scd1* and *scd5* genes were about 4000 bp and 1790 bp, respectively. Only the broadly conserved sites for miRNA families among vertebrates were considered in this study. The predicted miRNAs were then introduced to the miR2Disease Base (http://www.mir2disease.org/) to establish the relationship between miRNAs and human diseases.

### 2.3. Relative Rate Test

The substitution rates of the *scd* genes were compared among different paralogs inferred from the phylogenetic tree using the relative rate test implemented in RRTree [23]. Three phylogroups were defined as vertebrate *scd1*, vertebrate *scd5*, and invertebrate *scd* gene. The yeast *ole1* gene was used as outgroup. 

### 2.4. Selective Pattern Analysis

The ratio of synonymous substitution to nonsynonymous substitution (*ω* = *dN*/*dS*) is a good indicator to estimate the evolutionary selective pressure of protein-coding regions. The ratio of *ω* = 1, <1, and >1 indicates a neutral selection, a purifying selection, and positive selection, respectively. The *ω* ratios between pairwise sequences were estimated following the method of Yang and Nielsen [24]. 

The codon-substitution models were implemented using CODEML in PAML package [25]. All models fixed the transition/transversion rate and codon usage biases (F3×4). To determine the evolutionary selective patterns of two *scd* genes, the branch-specific model was applied to the data, which assumed that the foreground clade had different ratios from the background clade [26]. In model B, *scd1* and *scd5* genes were set as the foreground clade. In model C, *scd1*, *scd5,* and the invertebrate SCD homologs were set as three clades. In addition, we also determined the sites evolving under positive selection in a specific clade with the branch-site model that allows variation in *ω* across individual codons on a specific lineage [27]. We applied the modified branch-site model A (test 1 and test 2) [27], which permits variation of the *ω* ratio both among sites and lineages. The likelihood ratio tests (LRTs) were constructed to compare the fit to the data of two nested models. The significant difference between two models was evaluated by calculating twice the log-likelihood difference, and followed an *χ* ^2^ distribution with the number degree of freedom equal to the difference in the number of free parameters. 

## 3. Results

### 3.1. The Sequence Characteristics of SCD Orthologs

**Figure 1:**
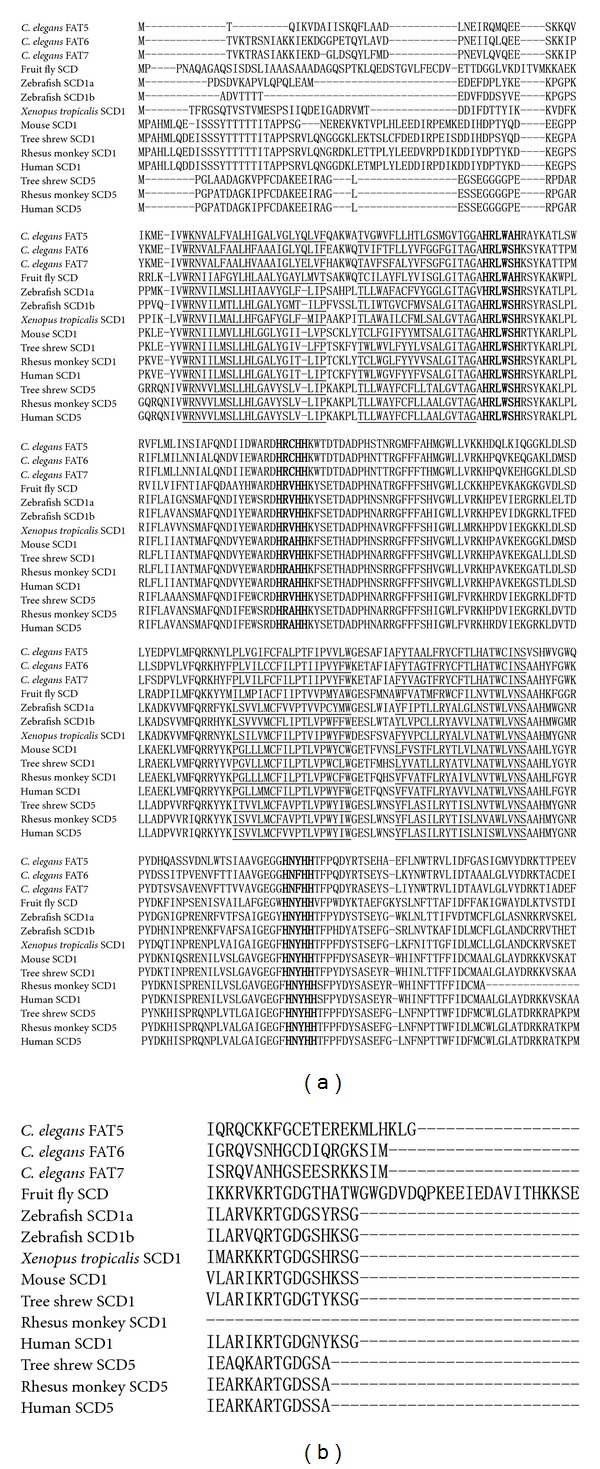
Alignment of inferred SCD protein sequences from 8 model animals. The three histidine motifs are in bold, and the four transmembrane hydrophobic domains were marked underline.

**Figure 2:**
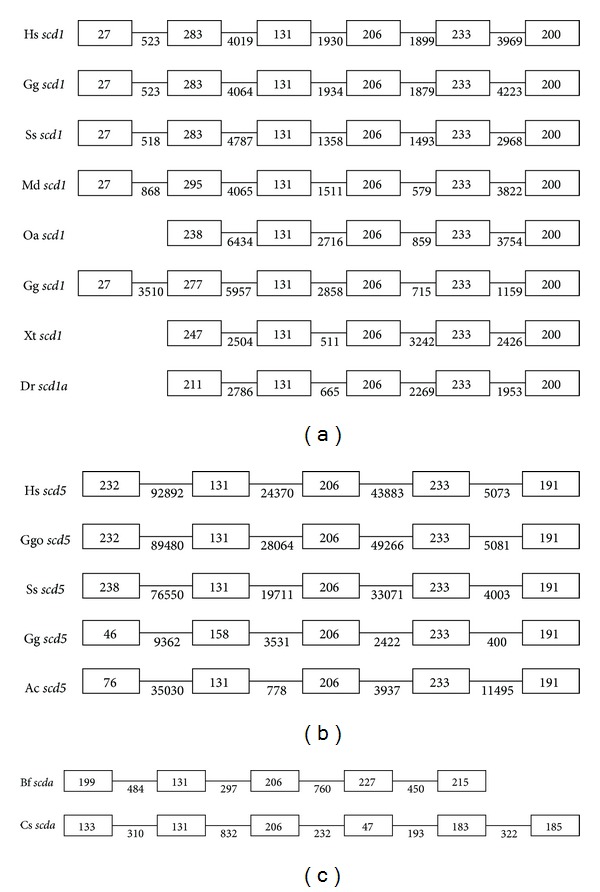
The exons size changes of *scd* genes. (a) Exon size changes of *scd1* gene in vertebrates. (b) Exon size changes of *scd5* gene in vertebrates. (c) Exon size changes of *scd* genes in invertebrates. Numbers in box represent the sizes of exons and numbers under bars represent the sizes of introns. Hs, *Homo sapiens*; Ggo, *Gorilla gorilla*; Ss,* Sus scrofa*; Md, *Monodelphis domestica*; Oa,* Ornithorhynchus anatinus*; Gg, *Gallus gallus*; Xt, *Xenopus tropicalis*; Dr, *Danio rerio*; Ac, *Anolis carolinensis*; Bf, *Branchiostoma floridae*; Cs, *Ciona savignyi*.

In human, the size of *scd1* gene is about 17 kb and 170 kb for* scd*5 gene. Though the remarkably different sizes of two *scd* genes, the full lengths of both *scd* encoded proteins are very close that SCD1 has 359 aa and SCD5 330 aa (Figure 1). To determine the conservation of SCD orthologs, we first investigated the sequence characteristics of SCD proteins. Comparison of the SCD amino acid sequences from several animal organisms revealed that the three histidine motifs HRLWSH, HRAHH, and HNYHH that exist in human SCD are also highly conserved in all alignments (Figure 1). But, the three histidine motifs also display minor changes in some organisms. For example, HRLWAH exists in *C. elegans* FAT-5 and *Drospholia* SCD genes; HNFHH in *C. elegans* FAT-6 and FAT-7 (Figure 1). The four transmembrane hydrophobic domains marked underline are also conserved in all alignments (Figure 1). Then, we investigated the sizes and order of exons of *scd* genes in several representative eukaryote organisms (Figure 2). Most of the *scd1* genes (e.g., chicken, human, etc.) are consisted of 6 exons. However, some vertebrate* scd1* genes only have 5 exons, like platypus and zebrafish. All of the *scd5* genes are consisted of 5 exons. Very interestingly, except the exon 1, the sizes and order of other exons (exon 2 (131), exon 3 (206), exon 4 (233), and exon 5 (191)) of *scd5* genes were not only separately equal but also very similar to the sizes and order of the third to sixth exons of *scd1* genes (exon 3 (131), exon 4 (206), exon 5 (233) and exon 6 (200)) in eukaryote organisms (Figures 2(a) and 2(b)). 

### 3.2. Phylogenetic Inference of *scd* Gene Lineages

**Figure 3:**
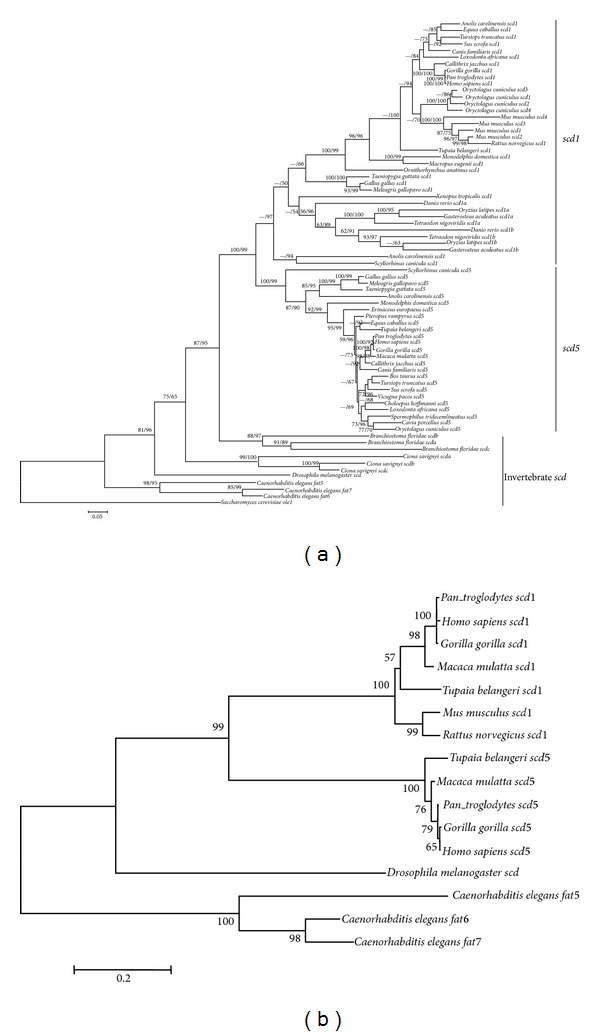
Phylogenetic trees of eukaryote *scd* isoforms. (a) Phylogenetic trees based on the nucleotide sequence data. The numbers on nodes indicated the support values, the former number was calculated using PHYML, and the latter number was calculated by FastTree 2.1.3. If bootstrap values were less than 50%, they were defaulted. Trees were rooted by yeast *ole1* gene. (b) Phylogenetic trees based on the amino acid sequences of 9 model animals with MEGA 4.0. The numbers on nodes indicated the support values. If bootstrap values were less than 50%, they were defaulted. Trees were rooted by *C. elegans *SCD paralogs.

The phylogenetic tree of *scd* genes based on the 73 nucleotide sequences from 39 species is shown in Figure 3(a) (TreeBASE Accession URL http://purl.org/phylo/treebase/phylows/study/TB2:S14739). The *scd* orthologs of invertebrate species are placed at the base of the tree using *scd* ortholog yeast* ole1* as outgroup. The *C. elegans fat-5*, *fat-6*, and *fat-7* are placed at the most basal position of the tree. In addition, the *scd1a, scd1b*,and* scd1c* from *Ciona savignyi* and amphioxus *Branchiostoma floridae* are just located out of the vertebrate lineages. Intriguingly, the *scd* genes in vertebrates are split into two lineages with strong support (support  value = 99%) according to the *scd* gene classification, suggesting that independent duplication events occurred in vertebrates after separation from invertebrates during evolution. In teleost fish, two *scd1 *paralogs were also diverged into two independent clades with high support, but the *scd5* gene was lost. This evolutionary pattern might suggest that the teleost fish *scd* experienced an ancient gene duplication event [12] or the genome duplication [6]. 

### 3.3. Evolutionary Rates and Selective Pattern in *scd* Genes

To determine whether the paralogs of *scd* evolve at the similar rates, the relative rate analysis was performed among *scd* gene and in which the invertebrate *scd* genes, vertebrate *scd1* and *scd5* genes were separated into 3 groups using the yeast *ole1* as outgroup. The analysis revealed that the *scd* genes were evolved at the similar evolutionary rate (*P* < 0.05). 

To address the selective constraint pattern within *scd* genes, the ratios of nonsynonymous (*dn*) to synonymous (*ds*) were estimated between two sequences. The analysis suggested that nearly almost pairwise comparisons of *scd* genes had a *ω* < 1, indicating a strong purifying selection. Intriguingly, the pairwise comparisons among *scd1* genes of human, gorilla, and chimpanzee had a *ω* = *∞*, which might result from that the nonsynonymous substitution occurred while the synonymous substitution did not in *scd1* sequences probably because of the very close relationships among these three species.

The selective pattern of *scd* genes was further performed using the condon-based maximum likelihood analysis (Table 1). In this analysis, the yeast *ole1 *was excluded. The estimated one ratio of *ω*_0_ (0.08684) over all sites and branches from the *scd* genes was substantially smaller than 1, suggesting a strong purifying selection (Table 1). In the branch-specific models, Model B assumes *scd1* gene and *scd5* gene as the foreground clades, respectively. In this model the estimated *ω* value was 0.09207 for *scd1 *gene and 0.07951 for the background clades. The estimated *ω* value was 0.06146 for *scd5 *gene and 0.09735 for the background clades. The LRT test suggested that the two-ratio model was not fit for the data better than the one-ratio model for *scd1* gene (*P* > 0.05) but fit better for *scd5* gene (*P* < 0.001). Under Model C, *ω* estimates for *scd1*, *scd5,* and invertebrate *scd* gene were 0.06140, 0.09198, and 0.11788, respectively. The LRT test indicated that Model C was significantly better fit for the data than did the one ratio model (M0) (*P* < 0.001). 

In addition, we determined the amino acid sites under positive selection at SCD1 and SCD5 clades on the phylogeny using the branch-site model. In this model, the SCD1 and SCD5 clades were assumed as the foreground clades, respectively. As seen in Table 1, the results of test 1 analysis designated several amino acid sites under the relaxed selection (*P* < 0.001) in both the *scd1* and *scd5* genes. However, none of the LRT test for *scd* genes was significant in test 2 analysis, indicating that the null hypothesis of the test 2 could not be rejected in both of the *scd* genes, and none of the two *scd* genes was underrelaxed selective constraint or under positive selection. Thus, we did not find any evidence for positive selection in both of the *scd* genes under these analyses.

### 3.4. The Regulation Analysis of *scd* Genes

Numerous transcription factors, for example, SREBP1, LXR, PPAR*α*, C/EBP-*α*, NF-1, NF-Y, and Sp1, have been revealed to bind to the *scd1* promotor region [13]. The consensus binding sites for the SREBP1, PPAR-*α*, C/EBP-*α*, NF-1, and NF-Y were known to mediate the insulin response, whereas the binding sites for Sp1 and AP1 were known to be the leptin response element. To determine whether these transcription factors also bind to *scd5* promotor region, the transcription binding site prediction was performed by using TRANSFAC and Promo. C/EBP-*α*, AP1, SP1, NF-1, NF-Y, and SREBP1 were detected at the promoter region of *scd5* gene of four species (Table 2). But SREBP1 was not detected in the promoter region of *scd5 *gene in other mammals (results not shown). Because SREBPs are weak transcriptional activators on their own, they interact with their target promoters in cooperation with additional regulators, most commonly including one or both of the transcription factors NFY and SP1 [28–30], and their binding sites were possessed a high degree of overlap [31]. We also detected the binding sites of NFY and SP1 near the binding site of SREBP1 in human. In this analysis, we detected the binding site of PPAR*α* by Promo, but not by TRANSFAC. However, the binding site of PPAR*α* detected in *scd5* gene was different from that of in *scd1* gene (Table 2). Though most of the transcription factor binding sites in *scd1* gene could be detected in *scd5* gene, the regulation of these transcription factors on *scd5* gene still needs further experimental verification.

**Figure 4:**
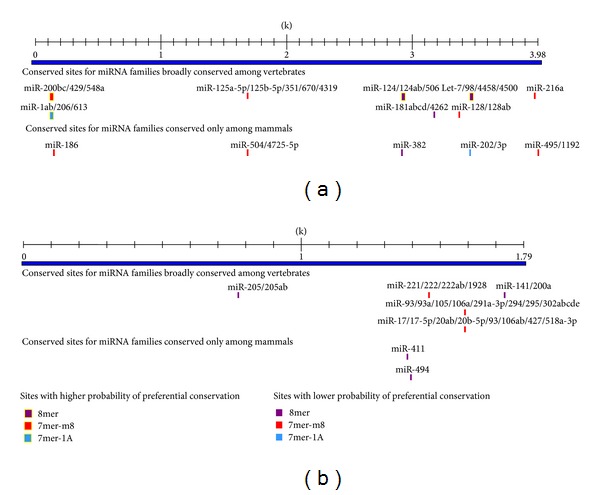
The target sites for miRNA families conserved among mammals and vertebrates at the 3′-UTR region of human* scd1* gene (a) and* scd5* gene (b). The sites with different probability of preferential conservation were marked in different colors. The target sites sharing among miRNAs separated by slash were marked with same color.

In order to compare the microRNAs regulation on *scd* genes, we predicted the microRNA target sites at the 3′-UTR region of *scd1* and* scd5* genes using TargetScan. The lengths of 3′-UTR region of* scd1* and* scd5* gene were about 4000 bp and 1790 bp, respectively (Figure 4). Within the 3′-UTR region of* scd1* gene, 8 conserved sites of microRNA families were predicted among vertebrates and 5 conserved sites were predicted among mammals (Figure 4(a)). Among these 13 microRNA families, almost all of them were closely associated with the cancers, for example, the miR-128, Let 7, miR-206, and miR-124a linked to breast cancer [32–35], hepatocellular carcinoma [36–38], and pancreatic cancer [39, 40]. In addition, plenty of evidence has described that the *scd1* acted as a potential target to prevent or treat metabolic syndrome. Among these microRNA families, several microRNAs were associated with the nonalcoholic fatty liver disease (NAFLD), type 2 diabetes, and diabetic nephropathy; for example, miR-429 and Let 7cde were closely related to NAFLD [41, 42]; miR-181a related to diabetes [43]; miR-216a related to diabetic nephropathy [44]. At the 3′-UTR region of* scd5* gene, 5 conserved sites of microRNA families were predicted among vertebrates and 2 conserved sites were predicted among mammals (Figure 4(b)). All of these microRNA families were closely associated with cancers.* scd5* gene was mainly expressed in brain and pancreas. Several microRNAs were associated with the neurological disorder and pancreatic cancers. miR-106a was associated with autism [45], miR-17 with glioma [46], miR-20b with schizophrenia [47]. miR-205, miR-221, miR-222, miR-17-5p, and miR-20a were associated with pancreatic cancers [39, 48–50]. Only 2 microRNAs, miR-200ab and miR-17, were linked to NAFLD [41, 42]. 

## 4. Discussion

The phylogenetic trees show that homologs of* scd *gene from invertebrates were all placed at the basal position of the tree, whereas the *scd* genes in vertebrates were diverged into two independently duplicated genes early in vertebrate evolution with strong support, in which all *scd1* genes form a distinct clade and all *scd5* genes clustered into another clade (Figures 3(a) and 3(b)). Our phylogenetic analysis was consistent with the previous studies by Castro et al. [6] and Lengi and Corl [11]. This pattern of duplication might be resulted from part of the two rounds of genome duplication in vertebrate ancestry [6]. 

When a gene duplication event occurs, the duplicated genes have redundant functions. The fate of the duplicated genes might be loss of function, gaining a new function, or subfunctionalization [51]. Subfunctionalization occurred when both duplicates can be stably maintained in the genome [52]. The division of gene expression after gene duplication appears to be a general form of subfunctionalization [53, 54]. In this model, after gene duplication, complementary degenerate mutations are fixed randomly underrelaxed functional constraints [55]. Previous studies suggested that both *scd1* and *scd5* encode the same functional delta-9 desaturase and are localized on endoplasmic reticulum (ER) membrane [3, 56]. However, the *scd1* gene expressed ubiquitous, and *scd5* gene expressed mainly in brain in different species [3, 6, 11]. We inferred that the evolution of *scd* genes might be a division of gene expression subsequent to gene duplication. This pattern was supported by the evolutionary forces behind the expression division of duplicate genes. The relative rate test suggested that the two duplicated *scd* genes evolved at the similar rate. The selective constraints analysis suggested that the *scd1* and *scd5* were both under strict purifying selection (Table 1), which was consistent with the conserved delta-9 desaturase of both *scd* genes. Intriguingly, in the branch-site analysis, we detected that some sites within *scd1* and *scd5* were underrelaxed selective pressure. These sites might be resulted from the random fixation of the complementary degenerate mutations that were underrelaxed functional constraints. 

Though both of *scd1* and *scd5* encoded delta-9 desaturase, producing a palmitoleic acid (16:1n7) and oleic acid (18:1n9) [3, 56], they expressed diversely in the physiological process. Previous studies had proposed that *scd1* were associated with a variety of diseases including cancers, type 2 diabetes, and cardiovascular disorders [13], whereas *scd5* might act a potential role for maintaining the optimum levels of oleic acid in brain development and physiological activities [3, 57]. Castro et al. proposed that the major distinction between* scd5 *and *scd1* would be at the regulatory level, in which *scd1* gene expression was mainly modulated at the transcriptional level by a wide variety of hormones and nutrients, whereas *scd5* was not responsive to external inputs like food sources [6]. In this study, we predicted the transcription factor binding sites at the 5′-UTR region and the miRNA target sites at the 3′-UTR region of human* scd5* gene. The transcription factor binding sites detected in *scd1* gene [13] could also be detected in *scd5* gene. However, the SREBP1 binding site only presents in human *scd5* gene, but not in other mammals, for example, rhesus monkey, pig and others. This might be that the prediction of transcription factor (TF) binding sites was based on known TF binding sites so that some new TF binding sites can not be detected. Recent studies have suggested that SREBP1 regulates the expression of *scd5* in primary cultures of human skeletal muscle cells [58], or directly binding to the promoter region of *scd5* in bovine [59]. In contrast, a study on human hepatocyte cell line suggested that SREBP1 only binds to the *scd1* gene, but not to *scd5* gene [31]. This discrepancy might be the distinct expression of *scd5* gene in different species or tissues. From our prediction, we conclude that the TF binding sites predicted in *scd5* gene were very similar to these of *scd1* gene, suggesting that the regulators may also be similar between two *scd* genes. Certainly, these TF predictions need further experimental verification. 

miRNAs regulation is another gene regulatory mechanism in posttranscriptional regulation. Gu et al. estimated the time of vertebrate miRNA duplication events and suggested that gene/genome duplications in the early stage of vertebrates may expand the protein-encoding genes and miRNAs simultaneously [60]. Gene duplication events, followed by subfunctionalization and neofunctionalization processes, are considered to be a major source for emergence of novel miRNA genes [61]. In this study, the lengths of 3′-UTR of *scd1* and* scd5* gene are about 4000 bp and 1790 bp, respectively (Figure 4). A previous study suggested that genes with longer 3′-UTRs are regulated by more distinct types of miRNAs [62]. In our analysis, 13 miRNAs targeting sites are detected in the 3′-UTR of* scd1* gene, while 7 miRNAs targeting sites are detected in the 3′-UTR of* scd5* gene. Additionally, the length changes of 3′-UTRs in these two *scd* genes might suggest a differentiation of the regulatory mechanisms. miRNAs predicted to target the 3′-UTR region of *scd1* gene are associated with breast cancers, hepatocellular carcinoma, and metabolic syndromes such as diabetes, NAFLD. However, most of the miRNAs predicted to target the 3′-UTR region of *scd5* gene are related to the neurogenic disease and pancreatic cancer; and only 2 microRNAs are associated with the NAFLD. This regulatory pattern might be due to the high expression of* scd5* gene in brain and pancreas [3]. Additionally, a recent study has reported that the *scd5* gene plays a key role in the regulation of the neuronal cell proliferation and differentiation [56]. These results might indicate that the expression of *scd5* is implicated in brain development and physiological activity.

In addition, we also investigated the size and order of exons of *scd* genes. We found that the *scd1* gene has an extra exon (exon1) compared to *scd5 *gene (Figure 2). The first 45 amino acids of SCD1 were highly different from those of SCD5 (Figure 1). Though there is no histidine domain and transmembrane domain exists in this part of SCD1, about 30 residues constitute a motif responsible for the rapid degradation of SCD [63]. This result indicated that the degradation of two SCD might be very different. However, due to no information on the degradation of SCD5, the evolutionary changes of regulation on both *scd* genes and SCD proteins still need further investigation.

## 5. Conclusion

In summary, this study of evolutionary pattern of *scd* genes showed that *scd1* and *scd5* genes emerged due to the duplication event as well as that they may play different roles. We also detected that the *scd* genes were evolved at the similar rate and were under strictly purifying selection, consistent with the conserved function of delta-9 desaturase of both SCD. Furthermore, our study revealed several potentially adaptive amino acid changes, which might be resulted from the random fixation of the complementary degenerate mutations underrelaxed functional constraints. The prediction of transcriptional factor binding sites at the 5′-UTR and miRNAs at 3′-UTR of *scd* genes suggested that the regulators of *scd5* may be different from *scd1* gene, supportting the differentiation at the regulatory levels between* scd5 *and *scd1*. These findings increase the current knowledge of evolutionary patterns and function of *scd* genes in eukaryote. Yet, further experimental investigations need to elucidate the regulation and function of *scd* genes, especially the *scd5* gene.

## Supplementary Material

Supplementary Table: Information of species, genes and sequence code used in this study.

## Figures and Tables

**Table 1:** Selective patterns of scd genes estimated in CODEML.

Model	ln *L*	Parameters estimates	2Δ*L*	Positively selected sites
Branch-specific models				
M0	−20340.727636	*ω* = 0.08684		
Model B				
*Scd1* two ratio	−20339.045042	*ω*_0_ = 0.09207, *ω*_1_ = 0.07951	3.365188	
*Scd5* two ratio	−20318.455800	*ω*_0_ = 0.06146, *ω*_1_ = 0.09735	44.542672^###^	
Model C Three ratio	−20315.506084	*ω*_*scd*1_ = 0.06140 *ω*_*scd*5_ = 0.09198 *ω*_invertebrate *scd*_ = 0.11788	50.443104^###^	

Branch-site models				
*Scd1 *				
Model A1	−20079.883939	*ω*_0_ = 0.06891, *ω*_1_=1, *ω*_2_ = 1 *P*_0_ = 0.85305, *P*_1_ = 0.04177		
M1a	−20178.290653	*ω*_0_ = 0.07950, *ω*_1_ = 1 *P*_0_ = 0.91928, *P*_1_ = 0.08072	196.813428^###^	
Model A	−20079.883939	*ω*_0_ = 0.06891, *ω*_1_ = 1, *ω*_2_ = 1 *P*_0_ = 0.85305, *P*_1_ = 0.04177	0	108L**, 109F**, 201A**, 206S, 212K**, 247Y**, 254A, 255I*, 276K**, 289V*, 315P, 330Y, 339A
*Scd5 *				
Model A1	−20129.099054	*ω*_0_ = 0.07500, *ω*_1_ = 1, *ω*_2_ = 1 *P*_0_ = 0.88996, *P*_1_ = 0.05968		
M1a	−20168.513281	*ω*_0_ = 0.07951, *ω*_1_ = 1 *P*_0_ = 0.91900, *P*_1_ = 0.08100	78.828454^###^	
Model A	−20129.099054	*ω*_0_ = 0.07500, *ω*_1_ = 1, *ω*_2_ = 1 *P*_0_ = 0.88996, *P*_1_ = 0.05968	0	157A**, 194P**, 215M**, 223P, 230I, 338A**

^###^ *P* < 0.001; ^##^0.001 < *P* < 0.01;

**P > 0.99; *P > 0.95.

**Table 2:** Transcription factor binding sites predicted at the 5′UTR of *hscd5. *

Transcription factor	Binding sites	Position (*hscd1*)^$^	Position (*hscd5*)
C/EBP*α*	GMAAA	−219	−1061, −1648
AP1	TGACC	−204, −271	−580, −643
SP1	GGCGG	−304, −314, −551	−286, −946
NF-Y	CCAAT	−458, −501	−397, −976
NF-1	TTGGC	−459, −502	−395
SREBP1	TCACC	−517	−892*
PPAR*α*	AAAG/GGTCA	−1186	−579^#^
T3R	GGTCA	−2228	−1223, −2245

T3R: tri-iodothyronine receptor; AP1: activator protein 1; NF-1/Y: nuclear factor 1/Y; SREBP1: sterol regulatory element binding protein; PPAR*α*: peroxisome proliferator-activated receptor; C/EBP*α*: CAAT/enhancer binding protein.

^$^These transcription factor binding sites were from [64].

^#^Predicted by PROMO.

*Only found in human using TRANSFAC.
