# Supplementary material for: The Evolutionary Pattern and the Regulation of Stearoyl-CoA Desaturase Genes
Source: Biomed Res Int. 2013 Nov 7;2013:856521. doi: 10.1155/2013/856521 (PMC3838806; doi:10.1155/2013/856521)
Supplement: Supplementary file 1 — Supplementary Table: Information of species, genes and sequence code used in this study. [file 856521.f1.pdf]

Table S1. Information of species, genes and sequence code used in this study

Species	Genes	Sequence code
Saccharomyces cerevisiae	*ole-1*	Ensemble: YGL055W
Caenorhabditis elegans	*fat5*	WormBase: WBGene00001397
	fat6	WormBase: WBGene00001398
	fat7	WormBase: WBGene00001399
Drosophila melanogaster	*hsc70-4，scd*	FlyBase: FBgn0001219
Ciona savignyi	*scda*	Ensemble: ENSCSAVP00000003593
	scdb	Ensemble: ENSCSAVP00000004039
	scdc	Ensemble: ENSCSAVP00000003934
Branchiostoma floridae	*scda*	GenBank: XP_002588865
	scdb	GenBank: XP_002585987
	scdc	GenBank: XP_002596094
Danio rerio	*scd1a*	ZFIN: ZDB-GENE-031106-3
	scd1b	ZFIN: ZDB-GENE-050522-12
Gasterosteus aculeatus	*scd1a*	Ensemble: ENSGACG00000002397
	scd1b	Ensemble: ENSGACG00000008487
Oryzias latipes	*scd1a*	Ensemble: ENSORLG00000009222
	scd1b	Ensemble: ENSORLG00000006839
Tetraodon nigoviridis	*scd1a*	Ensemble: ENSTNIG00000019395
	scd1b	Ensemble: ENSTNIG00000000140
Xenopus tropicalis	*scd*	Ensemble: ENSXETG00000023757
Scyliorhinus canicula	*scd1*	GenBank: JF729408
	scd5	GenBank: JF729409
Anolis carolinensis	*scd1*	Ensemble: ENSACAP00000010645
	scd5	Ensemble: ENSACAP00000010271
Taeniopygia guttata	*scd1*	Ensemble: ENSTGUP00000007924
	scd5	Ensemble: ENSTGUP00000002919
Gallus gallus	*scd1*	Ensemble: ENSGALP00000039331
	scd5	Ensemble: ENSGALP00000018194
Meleagris gallopavo	*SCD1*	Ensemble: ENSMGAG00000007446
	SCD5	Ensemble: ENSMGAG00000008016
Ornithorhynchus anatinus	*scd1*	Ensemble: ENSOANG00000002477
Monodelphis domestica	*scd1*	Ensemble: ENSMODG00000010632
	scd5	Ensemble: ENSMODG00000011983
Macropus eugenii	*scd1*	Ensemble: ENSMEUG00000004515
Mus musculus	*scd1*	Ensemble: ENSMUSG00000037071
	scd2	Ensemble: ENSMUSG00000025203
	scd3	Ensemble: ENSMUSG00000025202
	scd4	Ensemble: ENSMUSG00000050195
Rattus norvegicus	*scd1*	GenBank: NP_631931
Tupaia_belangeri	*scd1*	This study
	scd5	

Oryctolagus cuniculus	*scd1*	GenBank: XP_002718695
	scd2	GenBank: XP_002718696
	scd3	GenBank: XP_002718697
	scd4	GenBank: XP_002718662
	scd5	Ensemble: ENSOCUG00000005937
Macaca mulatta	*scd1*	Ensemble: ENSMMUP00000014179
	scd5	Ensemble: ENSMMUG00000000866
Gorrila gorrila	*scd1*	Ensemble: ENSGGOP00000001081
	scd5	Ensemble: ENSGGOP00000004994
Pan_troglodytes	*scd1*	Ensemble: ENSPTRG00000002847
	scd5	Ensemble: ENSPTRG00000016220
Homo sapiens	*scd1*	Ensemble: ENSG00000099194
	scd5	Ensemble: ENSG00000145284
Loxodonta africana	*scd1*	Ensemble: ENSLAFP00000014914
	scd5	Ensemble: ENSLAFP00000013895
Canis familiaris	*scd1*	GenBank: Xp543968
	scd5	GenBank: Xp544953
Tursiops_truncatus	*scd1*	Ensemble: ENSTTRP00000011286
	scd5	Ensemble: ENSTTRP00000007215
Sus scrofa	*scd1*	Ensemble: ENSSSCG00000010554
	scd5	Ensemble: ENSSSCG00000009245
Equus caballus	*scd1*	Ensembl: ENSECAG00000014658
	scd5	Ensembl: ENSECAT00000015537
Vicugna pacos	*scd1*	Ensemble: ENSVPAP00000009395
	scd5	Ensemble: ENSVPAP00000002239
Pteropus vampyrus	*scd5*	Ensemble: ENSPVAG00000016815
Bos taurus	*scd5*	Ensemble: ENSBTAT00000025820
Callithrix jacchus	*scd5*	Ensemble: ENSCJAG00000017338
Spermophilus tridecemlineatus	*scd5*	Ensemble: ENSSTOG00000006758
Cavia porcellus	*scd5*	Ensemble: ENSCPOP00000002936
Choloepus hoffmanni	*scd5*	Ensemble: ENSCHOP00000003699
Erinaceus europaeus	*scd5*	Ensemble: ENSEEUT00000011642
